# Socioeconomic Inequalities in Oral Health-Related Quality of Life among Brazilians: A Cross-Sectional Study

**DOI:** 10.3390/dj7020039

**Published:** 2019-04-02

**Authors:** Fabíola Bof de Andrade, Flavia Cristina Drumond Andrade

**Affiliations:** 1 Oswaldo Cruz Foundation (FIOCRUZ), Rene Rachou Research Institute, Belo Horizonte 30190-009, Brazil; 2 University of Illinois at Urbana-Champaign, Department of Kinesiology and Community Health and School of Social Work, Urbana, IL 61801, USA; fandrade@illinois.edu

**Keywords:** oral health, inequalities, quality of life, socioeconomic status

## Abstract

Objective: Assess the magnitude of the socioeconomic inequalities related to the impact of oral health on quality of life among adults and elderly individuals. Methods: This was a cross-sectional study with data from the most recent oral health survey from the state of Minas Gerais, Brazil. The sample included data on 2288 individuals—1159 adults in the 35–44 age group and 1129 adults in the 65–74 age group. Socioeconomic inequalities in Oral Impacts on Daily Performance ratings were measured using two inequality measures: the slope index of inequality (SII) and the relative index of inequality (RII). Results: The prevalence of negative impact of oral health on quality of life was 42.2% for the total sample, 44.9% among adults and 37.5% among elderly individuals. Significant absolute and relative income inequalities were found for the total sample (SII −27.8; RII 0.52) and both age groups (adults: SII −32.4; RII 0.49; elderly: SII −18.3; RI 0.63), meaning that individuals in the lowest income level had the highest prevalence of negative impacts. Regarding schooling, no significant differences were observed among the elderly. Conclusion: There were significant socioeconomic inequalities related to the negative impact of oral health-related quality of life in Brazil among both age groups.

## 1. Introduction

Despite improvements in the average oral health status in many countries [1], the burden of oral health diseases is not equally shared within societies. Problems with oral health disproportionately affect poor and other disadvantaged populations [2]. Two major oral health problems are dental caries and tooth loss, both of which can be prevented [3]. Individuals with poor oral health have lower quality of life due to pain, discomfort and impaired oral functioning [4].

There is consistent evidence of social gradients on oral health [1,5]. Prevalence of tooth loss [5], dental caries and edentulism are higher among individuals with lower socioeconomic status around the world [1]. Subjective measures of oral health also highlight important health inequalities, with those with lower socioeconomic status reporting higher levels of negative impact on quality of life [6,7].

Oral health has a stronger impact on the quality of life among adults than among elderly individuals [8,9]. Nonetheless, results seem to vary across socioeconomic groups [8,10,11]. For instance, a previous study using data from the Brazilian National Oral Health Survey found that poor oral health had a greater impact on quality of life among adults with low education than those with greater education but there was no association among elderly individuals. Similarly, a study of people in the South of Brazil found no socioeconomic differences in the negative impact of oral health on quality of life among elderly [11]. However, a recent study [10] found that elderly Brazilian with low income had higher impact of oral health on quality of life.

Assessing the socioeconomic inequalities in oral health-related quality of life is important for the development and enhancement of public health policies. Most studies that have evaluated the association between socioeconomic factors and oral quality of life were conducted in developed countries. However, little is known about this association in developing countries, where oral health problems are more prevalent. Moreover, most studies to date have examined whether socioeconomic factors are associated with the impact of oral health on quality of life but they have not measured the magnitude of socioeconomic inequalities in oral health-related quality of life. We hypothesize that there were significant socioeconomic inequalities in the impact of oral health on quality of life among adults and older adults in Brazil. Thus, the aim of this cross-sectional study was to measure the magnitude of socioeconomic inequalities in the negative impact of oral health on quality of life in Brazil among adults and elderly individuals.

## 2. Materials and Methods

This was a cross-sectional study with data from the SB Minas Gerais Project conducted in 2012. This project is the most recent examination of the oral health conditions of the population residing in the state of Minas Gerais. With 21 million residents according to the 2010 census, Minas Gerais is among the most populous states in Brazil. The sample was representative of the state population. Study procedures and details on the sample selection have been published elsewhere [12].

Data collection was conducted in the household by oral surgeons trained for the task. Participants answered to a structured questionnaire containing questions related to behavioral and socioeconomic conditions. After answering the questionnaire, participants underwent a clinical oral health examination, which followed a standardized methodology stipulated by the World Health Organization [13]. The total sample included 2404 individuals and 65 (2.7%) did not have complete information on selected variables. The present study included data on 2288 individuals who had complete information—1159 adults in the 35–44 age group and 1129 in the 65–74 age group. These age groups are selected based on the recommendations by the World Health Organization to assess oral health in adults [13].

### 2.1. Outcome Measure

The Oral Impact on Daily Performance (OIDP) questionnaire was used to evaluate the impact of oral health on the quality. This instrument measures how oral health influences physical, psychological and social aspects of everyday life [14]. This measure has been included in the National Oral Health Surveys and SB Minas Gerais adopted it for comparability purposes.

Participants were asked if in the last 6 months they had difficulties performing the following activities: eating food; speaking clearly; cleaning teeth; performing physical activities; going out, studying or working; sleeping; smiling, laughing and showing teeth without embarrassment; maintaining usual emotional state without being irritable. Participants who answered “yes” to at least one of these questions were considered as having oral health negatively impacting quality of life.

### 2.2. Socioeconomic Position Indicator and Other Covariates

Schooling and family income were used as the socioeconomic position indicators. Schooling was based on years of formal schooling and was categorized into four groups: 0–3, 4–7 years (incomplete primary education), 8–11 years (complete primary to complete high school); 12+ years (higher education). Monthly family income was obtained during the interview using brackets of income in Brazilian currency (Reais) as follows: ≤500, 501–1500, 1,501–2500, 2501–4500, 4501 or more.

Other covariates included: demographic factors (age and sex) and clinical oral health measures (edentulism, functional dentition [presence of 20 or more teeth] [15], need for dental prostheses, use of dental prostheses and need for dental treatment).

### 2.3. Data Analysis

Initial data analysis included a description of the sample followed by a bivariate analysis between the dependent variable, socioeconomic position indicator and remaining covariates. We evaluated the association between the dependent variable and the covariates using the Rao-Scott chi-square test for complex samples. The description of the relationship between the oral health impact on quality of life was made using the equiplot (http://www.equidade.org/equiplot.php). In this graph each horizontal line presents the prevalence of the outcome according to the levels of the sociodemographic measures. It is possible to visualize the prevalence of the impact in each group and the distance between the groups, which represents the absolute inequality.

Socioeconomic inequalities in OIDP were measured using two inequality measures: the slope index of inequality (SII) and the relative index of inequality (RII). These indices are regression-based measures and take the entire socioeconomic distribution into account [16]. SII gives the absolute difference of the prevalence of the outcome between participants with the lowest education levels and those with the highest education level, whereas the RII provides the relative difference between these groups. Individuals were ranked cumulatively from 0 to 1 according to their educational level (income level) such that “0” represented the lowest education level (income level) and “1” represented the highest education level (income level). Each education category was assigned a ridit-score based on the midpoint of the range in the cumulative distribution of the population of participants in the given category. Values of SII lower than 0 and RII values lower than 1 indicate that individuals with lower education (income) are more likely to experience negative impact compared with those with more education (income).

The RII and SII were obtained by regressing the weighted score measure of education on OIDP. SII and RII were estimated, for each age group, using Poisson regression models [6,17]. All the models were adjusted for age, sex, use and need for dental prostheses, need for dental treatment and number of teeth. These variables were previously shown to be associated to the impact of oral health on quality of life in Brazil [8,10]. In all analyses, the significance level was set at 5% and a 95% confidence interval is provided. Statistical analyses were performed using STATA version 13.0 (StataCorp LP, College Station, TX, USA) using the “svy” command to account for the complex survey design.

### 2.4. Ethical Considerations

The SB Minas Gerais Project was approved by the Ethical Review Board of the Pontifical Catholic University of Minas Gerais (number 9173, 28 March 2012). All subjects gave their informed consent for inclusion before participating in the study.

## 3. Results

The prevalence of negative impact of oral health on quality of life was 42.5% [95% CI 38.3–46.9] for the total sample, 44.9% [95% CI 39.8–50.1] among the younger group and 37.5% [95% CI 32.4–42.8] among the older group (Table 1). For the total sample, about one fifth (19.3%) had 0–3 years of education. Among the younger group, most had eight or more years of schooling but a lower percentage of elderly individuals reported having this level of education (20.1%).

Table 2 shows the negative impact of oral health on quality of life according to socioeconomic and demographic variables.

**Figure 1:**
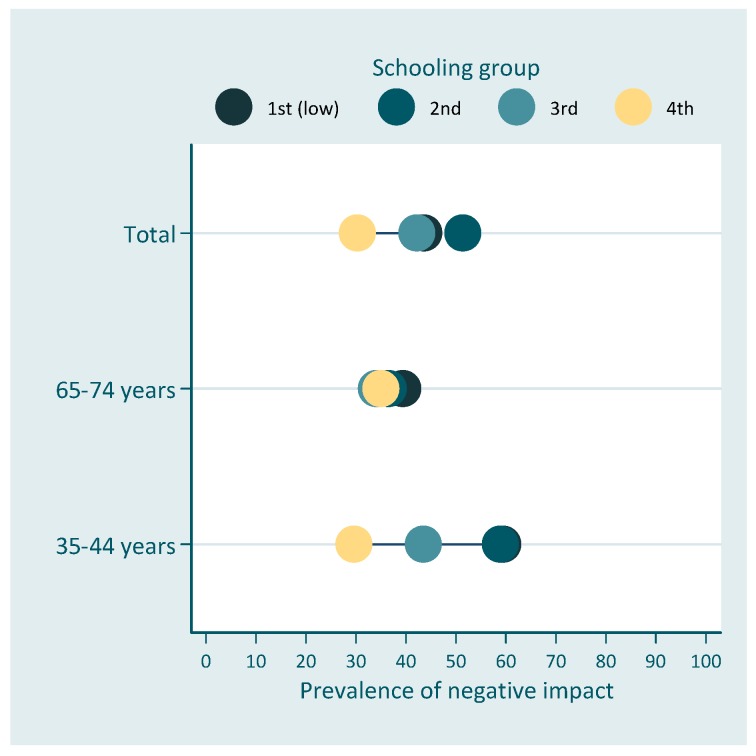
Prevalence of the negative impact of oral health on quality of life across education for the total population and among age-groups (equiplot). Schooling groups (in years of formal schooling): 1st = 0–3, 2nd = 4–7; 3rd = 8–11; 4th = 12+.

**Figure 2:**
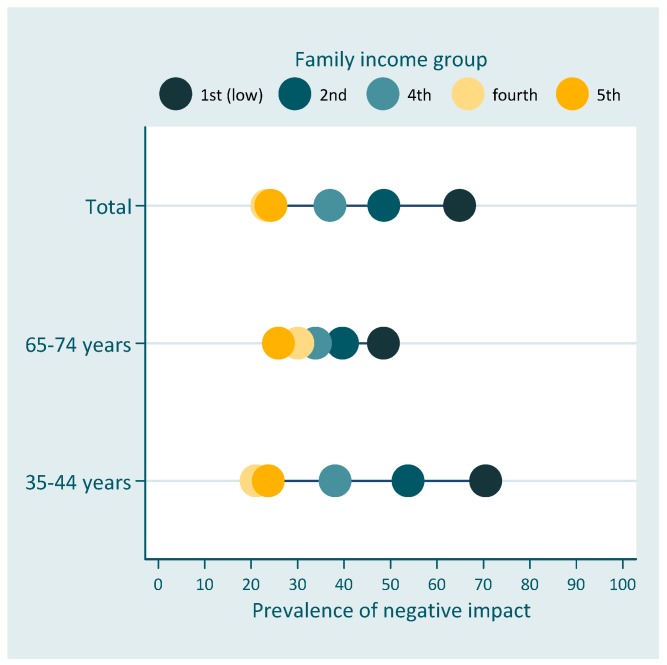
Prevalence of the negative impact of oral health on quality of life across family income for the total population and among age-groups (equiplot). Family income groups (in reais) = 1st = ≤500; 2nd = 501–1500; 3rd = 1501–2500; 4th = 2501–4500; 5th = 4501+.

Figure 1 and Figure 2 show the prevalence of negative impact of oral health on quality of life according to schooling and family income, respectively.

Results presented in Table 3 indicate absolute and relative inequalities related to family income on the impact of oral health on the quality of life for both age groups. Individuals in the higher income group had 27.8 percent-points lower prevalence than individuals in the lowest income rank. However, absolute inequalities were lower for elderly individuals [SII −18.3 (95% CI −34.5; −2.2)] than for the younger group [SII −32.4 (95% CI −47.4; −17.4)] (Table 3). Similar results were found for relative inequalities with the two groups, with lower socioeconomic position having more negative impact than higher socioeconomic position. No differences were found in relation to schooling. The prevalence of negative impact of oral health on quality of life was 52% lower among individuals in the highest income rank for the total OIDP in the total sample. Relative differences were smaller for elderly individuals [RII 0.63 (95% CI 0.40; 0.99)], than for adults [RII 0.49 (95% CI 0.34; 0.69)].

## 4. Discussion

The present study evaluated socioeconomic inequalities related to the impact of oral health on quality of life using a multidimensional instrument. To our knowledge, this was the first study to evaluate the magnitude of socioeconomic inequalities related to the oral health impact on quality of life in Brazil. The prevalence of negative impact was high, with 44.9% of younger adults and 37.5% of older ones reporting that their oral health had a negative impact in their daily activities in the 6 months prior to the survey. Significant absolute and relative income inequalities were found, with individuals in the lowest income group having the highest prevalence of negative impacts.

Our findings confirm previous studies that have identified higher prevalence of negative impact of oral health on quality of life among younger adults than among older adults in Brazil [18] and in other countries [19,20,21]. These differences across age groups may be associated with changes in expectations over the life course. It is possible that elderly individuals self-report better quality of life despite facing some difficulties in their daily activities [22]. In fact, Brazilian elderly individuals have a high prevalence of severe tooth loss and edentulism, which may have been acquired at younger ages. However, it is possible that they learn to adapt to these poor oral health conditions and adjust their perceptions and expectations related to their quality of life [22,23].

Regarding the magnitude of inequalities, the scope for comparison with other studies is limited, as only one study based on a sample from the United States and England [6] evaluated the magnitude of inequality related to the impact of oral health on quality of life using SII and RII. However, they used a different instrument to measure oral health-related quality of life—the Oral Health Impact Profile-14 [6]. In addition, that study focused on adults 25 years and older without differentiation as to age [6]. Based on the entire sample, the absolute income inequalities found in the present study was higher than the ones observed in the United States and England. However, relative inequalities in the present study were smaller.

Our findings confirmed previous studies conducted in Brazil that found no statistical association with education once results were adjusted for income [8,10]. This lack of association with education has been previously reported in different countries [24,25], including Brazil [8,26]. Among the reasons identified for the lack of association for education after adjusting for income may be the fact that income is more directly related to the ability to access and pay for dental treatment and rehabilitation services that can improve individuals’ quality of life. Education, which is generally completed in early adulthood, influences other socioeconomic conditions later in life, including income [27]. At older ages, educational level shows a weaker association with health than other material indicators, such as income [28,29].

Differences in the magnitude of socioeconomic factors are also related to cultural and behavioral experiences over the life course [23,25,30], which may explain the lower magnitude of inequalities among the elderly group. Thus, it has been suggested that age and cohort need to be observed while interpreting the relationship between oral health and socioeconomic factors [31]. New cohorts of elderly individuals have more teeth and have higher expectations in relation to their oral health [32]. As a result, inequalities may become wide in future cohorts of older adults as most of the tooth loss is likely to occur among those least privileged [33].

This study is based on a large representative sample of adults from the second most populous state in Brazil. Data used in the study came from an oral health survey, which made it possible to control for clinical variables that were previously reported to be important explaining oral health-related quality of life. The limitation of the present study is related to its cross-sectional design; therefore, we are unable to assess how education influences the impact of oral health over the life course and we cannot explore time-trends. Moreover, the study only used two measures of socioeconomic position and important variables such as access to health insurance were not collected in the study.

This study provides important evidence of the existence of inequalities in the impact of oral health on the quality of life among Brazilians adults and elderly individuals living in Minas Gerais, Brazil. The findings highlight the need to improve oral health throughout life and the need to reduce inequalities across socioeconomic groups.

## Figures and Tables

**Table 1:** Descriptive statistics (%) for the study variables.

		Age Groups	
	Total (*n* = 2288)	35–44 years (*n* = 1159)	65–74 years (*n* = 1129)
Sex			
Male	36.2	34.6	38.5
Female	63.8	65.4	61.5
Schooling (in years)			
0–3	19.3	6.0	47.6
4–7	30.7	30.0	32.0
8–11	26.6	33.7	11.4
12+	23.5	30.2	9.1
Family income (in reais)			
≤500	5.8	6.3	4.5
501–1500	52.5	48.4	61.3
1501–2500	25.5	27.6	20.9
2501–4500	11.8	12.9	9.4
4501+	4.4	4.7	4.0

**Table 2:** Negative impact of oral health on quality of life according to socioeconomic and demographic variables.

	Negative Impact		
	Total	35–44 years	65–74 years
	(%)	(%)	(%)
Sex			
Male	40.1 (34.6, 45.9)	41.8 (34.7, 49.3)	37.0 (30.3, 44.2)
Female	43.8 (39.0, 48.8)	46.5 (40.8, 52.4)	37.7 (31.5, 44.4)
Schooling			
0–3 years	43.6 (37.6, 49.8) *	59.4 (44.6, 72.7) *	39.4 (33.2, 45.9)
4–7 years	51.4 (45.0, 57.8)	58.9 (50.5, 66.8)	36.5 (30.0, 43.4)
8–11 years	42.2 (36.5, 48.2)	43.5 (37.3, 50.0)	34.1 (21.3, 49.8)
12+ years	30.3 (24.5, 36.8)	29.6 (23.4, 36.7)	35.0 (23.8, 48.2)
Family income			
≤500 reais	64.9 (55.0, 73.7) *	70.5 (58.3, 80.3) *	48.5 (31.4, 65.9)
501–1500 reais	48.6 (43.7, 53.5)	53.8 (47.2, 60.4)	39.7 (34.2, 45.5)
1501–2500 reais	37.0 (30.5, 44.0)	38.1 (30.6, 46.2)	33.9 (25.3, 43.6)
2501–4500 reais	23.4 (16.9, 31.5)	21.1 (13.9, 30.7)	30.1 (9.4, 43.6)
4501+ reais	24.3 (13.9, 39.0)	23.7 (10.3, 45.8)	25.9 (11.5, 48.3)

* *p* < 0.05.

**Table 3:** Absolute and relative inequalities related to the negative impact of oral health on quality of life according to the socioeconomic position measures.

	SII (95% CI)	RII (95% CI)
Schooling		
Total	−7.6 (−20.7; 5.6)	0.84 (0.61; 1.14)
35–44 years	−13.9 (−28.6; 0.7)	0.73 (0.53; 1.02)
65–74 years	3.0 (−13.4; 19.5)	1.14 (0.73; 1.77)
Family income		
Total	−27.8 (−39.2; −0.165) **	0.52 (0.39; 0.69) **
35–44 years	−32.4 (−47.4; −0.174) **	0.49 (0.34; 0.69) **
65–74 years	−18.3 (−34.5; −0.022) *	0.63 (0.40; 0.99) *

* *p* < 0.05; ** *p* < 0.001.
